# Accelerometer-derived physical activity, sarcopenia, and grip strength as modifiers of type 2 diabetes risk

**DOI:** 10.1038/s41598-026-54864-8

**Published:** 2026-05-28

**Authors:** Zhengqi Qiu, Yunzhi Emma Huang, Yu He, Juntao Kan, Lin Xu

**Affiliations:** 1 Center for Sleep and Circadian Medicine, The Affiliated Brain Hospital, Guangzhou Medical University, Guangzhou, 510370 Guangdong China; 2 Guangdong Engineering Technology Research Center for Translational Medicine of Mental Disorders, The Affiliated Brain Hospital, Guangzhou Medical University, Guangzhou, 510370 Guangdong China; 3 Amway R&D Center, Nutrilite Health Institute, 720 Cailun Road, Shanghai, 201203 China; 4 Renal Division, Department of Medicine, Institute of Nephrology, Peking University First Hospital, Peking University, Beijing, 100034 China; 5 School of Public Health, Sun Yat-sen University, No. 74, 2nd Zhongshan Road, Guangzhou, 510275 Guangdong China

**Keywords:** Type 2 diabetes mellitus, Sedentary behavior, Sarcopenia status, Grip strength, Accelerometer-based analysis, Diseases, Endocrinology, Health care, Medical research, Risk factors

## Abstract

Physical activity (PA) represents a modifiable protective factor against type 2 diabetes mellitus (T2DM), whereas sedentary behavior is recognized as a significant risk factor. However, uncertainties remain regarding the dose-response relationships across different PA intensities, the impact of sarcopenia status, and potential interactions between muscle function and PA. This prospective cohort study included 89,532 UK Biobank participants. PA and sedentary behavior were measured by wrist accelerometers. PA included total volume of PA, moderate-to-vigorous PA (MVPA), and light-intensity PA (LPA). Sarcopenia status was defined using EWGSOP2 criteria including grip strength, muscle mass, and walk pace. Cox proportional hazards models estimated associations with incident T2DM over median seven years of follow-up, adjusting for sociodemographic, lifestyle, and clinical factors. Higher levels of total PA and guideline-recommended MVPA were associated with reduced T2DM risk. Sedentary time showed a positive linear association (*P* for linearity < 0.001, *P* for nonlinearity = 0.969). A slow walking pace (HR = 1.82, 95% CI: 1.57–2.10) and severe muscle mass deficit (HR = 1.15, 95% CI: 1.00-1.32) were independent risk factors. Crucially, grip strength modified PA-T2DM associations: higher PA reduced risk significantly only in participants with normal grip strength (HR = 0.60, 95% CI: 0.53–0.69) but not in those with low grip strength (HR = 1.06, 95% CI: 0.74–1.51; *P* for interaction < 0.05). Accelerometer-measured PA demonstrated dose-dependent protective effects against T2DM, whereas components of sarcopenia were independently associated with an increased risk. Notably, preserved grip strength enhances these benefits, suggesting that muscle function assessment can optimize personalized prevention strategies.

**Supplementary Information:**

The online version contains supplementary material available at 10.1038/s41598-026-54864-8.

## Introduction

Type 2 diabetes mellitus (T2DM) is a major global health burden, with the affected population expected to rise to 1.31 billion by 2050.^1,2^ Although T2DM is already highly prevalent, its incidence continues to rise globally, probably due to environmental pollution^3^, dietary shifts^4^, and increasingly sedentary lifestyles^5^. Despite advances in pharmacological treatment, current approaches for T2DM remain reactive and insufficient to counter the rising incidence. Thus, identifying individuals at high risk of type 2 diabetes is crucial for effective primary prevention.

Several studies have found that physical activity (PA) is a key strategy for the prevention of T2DM^6,7^. In reality, PA exists on a continuous spectrum and can be categorized into moderate-to-vigorous intensity physical activity (MVPA) and light-intensity physical activity (LPA). Evidence of its dose-dependent effect remains uncertain, particularly concerning whether small increases in activity yield meaningful preventive benefits^8,9^. Sedentary behavior, representing the opposite end of the movement spectrum, has been associated with increased metabolic risk^10^. However, the extent to which sedentary behavior is associated with the risk of T2DM remains unclear. Importantly, methodological limitations, particularly the widespread reliance on self-reported PA data that are prone to recall bias and social desirability, have likely contributed to inconsistencies in the literature and hindered accurate effect estimation^11–13^. As highlighted by pivotal research, objective physical activity measures yield more robust risk estimates for cardiometabolic diseases compared with self-reported methods^14^. Employing objective PA assessment methods is therefore essential to accurately quantify dose–response relationships and clarify the long-term preventive impact of PA on T2DM, a concept increasingly supported by recent accelerometer-based prospective studies^15^.

The metabolic benefits of physical activity are fundamentally dependent on the functional capacity of skeletal muscle, which serves as the primary tissue for insulin-mediated glucose uptake^16^. Consequently, evaluating movement behaviors independently of an individual’s underlying muscle health may provide an incomplete assessment of their diabetes risk. Sarcopenia status, characterized by the progressive loss of muscle mass and physical function, is increasingly common among older adults^17,18^. Reduction in muscle mass may contribute to insulin resistance and impaired glucose metabolism^19^. While associations between muscle mass and T2DM have been examined in prior studies^20,21^, most relied on self-reported physical activity data that are prone to substantial recall bias, limiting the precision of dose-response relationships. Moreover, few studies have comprehensively assessed sarcopenia status (a combined phenotype of reduced muscle quantity and function) in relation to incident T2DM. Critically, it remains unknown whether sarcopenia components (particularly grip strength) modify the protective effect of objectively measured physical activity on T2DM risk.

Furthermore, grip strength acts as a straightforward and reliable proxy for overall muscle function and is a core diagnostic component of sarcopenia. Lower grip strength has been independently linked to increased risks of cardiovascular disease^22^ and T2DM^23^. Since muscular fitness dictates the capacity to engage in and physiologically respond to exercise, grip strength may not only act as an independent risk marker but also serve as a critical threshold in the pathway linking physical activity to glucose metabolism. Understanding this complex interplay between movement behaviors and muscular function could help refine risk stratification and inform personalized, multifaceted prevention strategies.

To address these gaps, this study leveraged large-scale accelerometer-derived data on PA and sedentary behavior from the UK Biobank. First, we aimed to investigate the association between sedentary behavior and different volumes of PA with the risk of developing T2DM. Second, we evaluated the association between sarcopenia status and the risk of T2DM. In addition, we explored whether grip strength modifies the association between PA and T2DM.

## Methods

### Study population

This study utilized data from the UK Biobank, a nationwide prospective cohort that enrolled over 500,000 participants aged 37–73 years between 2006 and 2010. Recruitment was conducted across 22 centers in the United Kingdom. All participants provided their consent for the utilization of their national health-related hospital and death records. The UK Biobank has received ethical approval from the National Health Service and the National Research Ethics Service (reference number: 11/NW/0382). Additional details on the study design were available in previously published sources.^24^.

### Accelerometer data collection and processing

Between February 2013 and December 2015, a total of 106,053 UK Biobank participants consented to wear a wrist-worn accelerometer (Axivity AX3, Axivity Ltd, York, UK) on their dominant hand for a continuous period of seven days. Of these, 103,682 valid datasets were obtained and included in the final analysis. The accelerometers were configured to record triaxial acceleration data at a sampling frequency of 100 Hz and within a dynamic range of ± 8 g^25,26^. Data calibration was performed by the UK Biobank accelerometer expert working group^24^, and periods of non-wear were identified using established protocols^25,26^. Further details on the processing procedures and quality control have been described previously^25,26^.

Total volume of PA was quantified as the average vector magnitude over a week, expressed in milli-gravity units (mg)^27^. Physical activity intensities were classified using a validated machine learning–based model, distinguishing between vigorous (VPA), moderate (MPA), and light physical activity (LPA)^28^. MPA and VPA were computed as the cumulative minutes per week in which five-second epochs met or exceeded acceleration thresholds of 100 mg and 400 mg, respectively^29^. LPA was defined as epochs with a mean acceleration ranging from 30 to 100 mg^30^, while sedentary behavior was characterized as any waking epoch with a mean acceleration below 30 mg^31^.

For analytical purposes, total PA, LPA, and sedentary behavior levels were stratified into tertiles (low, moderate, high). Moderate-to-vigorous physical activity (MVPA) was categorized based on the American Diabetes Association’s recommended threshold of ≥ 150 min per week, classifying individuals as meeting or not meeting PA guidelines^32^.

### Measurement of sarcopenia status

The presence of probable sarcopenia and sarcopenia were defined using the European Working Group of Sarcopenia in Older People 2 (EWGSOP2) definition for individuals of all ages.^33^ Participants were considered to have probable sarcopenia if they had low handgrip strength alone and confirmed sarcopenia if they also had low muscle mass. Sarcopenia was considered to be severe if low handgrip strength, low muscle mass and low physical performance were all present.

Hand grip strength: Hand grip strength was measured while sitting using a Jamar J00105 hydraulic hand dynamometer while sitting. The elbow of the arm holding the dynamometer was placed against the side of the body and bent to a 90° angle with the forearm placed on an armrest. Participants were instructed to squeeze the handle of the dynamometer as hard as they could for 3 s. Both left and right hands strengths were measured. The meaning of the right- and left‐side values, expressed as kg, was used in the analysis. The measured handgrip strength was further divided into sex-specific two groups: low (Female: <16.0 kg; Male: <27.0 kg), and normal (Female: ≥16.0 kg; Male: ≥27.0 kg)^34^.

Muscle mass: Low muscle mass is assessed using appendicular lean soft tissue (ALST), estimated from bioelectrical impedance analysis (BIA)-derived fat-free mass (FFM), using the equation developed by Dodds et al.: ALST (kg) = (0.958 × appendicular FFM [kg]) − (0.166 × S) − 0.308, where S is 0 for females and 1 for males.^34^ ALST primarily represents skeletal muscle, with minor contributions from skin and connective tissue; however, for simplicity, it is referred to as muscle mass throughout this study^35^. We defined the muscle mass deficit as: normal (ALST/height^2^ ≥7.98 kg/m^2^ for men and ≥ 6.15 kg/m^2^ for women, or ALST/BMI ≥ 0.94 for men and ≥ 0.64 for women), mild to moderate deficit (ALST/height^2^ < 7,98 kg/m^2^ for men and < 6.15 kg/m^2^ for women, or ALST/BMI < 0.94 for men and < 0.64 for women), and severe deficit (ALST/height^2^ < 6.95 kg/m^2^ for men and < 5.30 kg/m^2^ for women, or ALST/BMI < 0.84 for men and < 0.55 for women)^35^.

Physical performance: The UK Biobank did not contain an objective measure of gait speed, as specified by the European Working Group on Sarcopenia in Older People (EWGSOP) and the Foundation for the National Institutes of Health Sarcopenia Project (FNIHSP) criteria. As a surrogate marker of poor gait speed and low performance, we used walk pace^36^. The walking pace was self-reported via a touchscreen‐based questionnaire by answering the question “How would you describe your usual walking pace? (1) Slow pace, (2) Steady/average pace, and (3) Brisk pace”.

### Outcomes

The primary outcome of interest was incident T2DM, identified through hospital admission records or death registries, as detailed in Supplementary Table S1. Follow-up data were available through November 12, 2021. Participants were censored at the earliest occurrence of T2DM diagnosis, death, or the end of the follow-up period, whichever came first.

### Covariates

The following variables were potential confounding factors: age at the time of accelerometer wearing (continuous, years), sex (male/female), ethnicity (white/others), Townsend deprivation index (continuous, a score representing the deprivation of the participant’s neighbourhood as a reflection of their socioeconomic position), recruitment center (England/Wales/Scotland), educational level (degree or above/any other qualification/no qualification), season of accelerometer wear (spring/summer/autumn/winter), obesity status (underweight or normal weight/overweight/obese), healthy diet score (continuous), sleep duration (continuous, hours/day), grip strength (continuous, kg), smoking status (never/previous/current), alcohol consumption (not current/two or less times a week/three or more times a week), HbA1c (continuous, mmol/mol), hypertension (yes/no), high cholesterol (yes/no), history of coronary heart diseases (yes/no), history of cancers (yes/no), use of blood pressure-lowering medications (yes/no), and use of cholesterol-lowering medications (yes/no). Detailed information of covariates was described in Supplementary Table S1.

### Statistical analysis

Baseline characteristics across categories of moderate-to-vigorous physical activity (MVPA) were presented as counts and percentages for categorical variables, means with standard deviations (SD) for normally distributed continuous variables, or medians with interquartile ranges (IQR) for skewed data. Missing values for covariates were addressed using multiple imputation via the mice package in R. Details regarding the extent and pattern of missingness were provided in Supplementary Table S2. The imputation process was conducted under the assumption that missing data were not missing completely at random, acknowledging the potential influence of unobserved factors on data absence.

To examine the shape of the associations between total and intensity-specific PA, sedentary behavior, and incident T2DM, we conducted continuous dose–response analyses using restricted cubic spline models. Knots were placed at the 10th, 50th, and 90th percentiles to flexibly capture potential linear and nonlinear trends in the exposure–outcome associations.

Cox proportional hazards models were constructed to investigate the associations of PA, sedentary behavior, and sarcopenia status categories with the risk of incident T2DM. The results were presented as hazard ratios (HRs) and 95% confidence intervals (CIs). To assess potential effect modification, we examined the interactions between PA, sedentary behavior, and sarcopenia status using multivariable Cox regression models. Both the additive and multiplicative interaction terms were included in the model.

Additionally, to further explore potential effect modification, we conducted stratified analyses according to sarcopenia status to evaluate whether the associations between PA, sedentary behavior, and incident T2DM differed across sarcopenia subgroups. We repeated the main analyses within subgroups defined by age (< 65 years vs. ≥65 years), sex (female vs. male), and obesity status (underweight or normal weight, overweight, or obese) to examine whether the associations remained consistent across different populations. We conducted several sensitivity analyses to test the robustness of our findings. These included using Fine–Gray subdistribution hazard models to account for death as a competing risk for incident T2DM and repeating the analyses after excluding participants with missing covariate data or those who developed T2DM within the first two years of follow-up.

All Statistical analyses were performed using the R software Version 4.3.1 (R Development Core Team, Vienna, Austria). Statistical significance was set at *P* < 0.05 (two-sided test) was considered statistically significant.

## Results

### Baseline characteristics

**Fig. 1:**
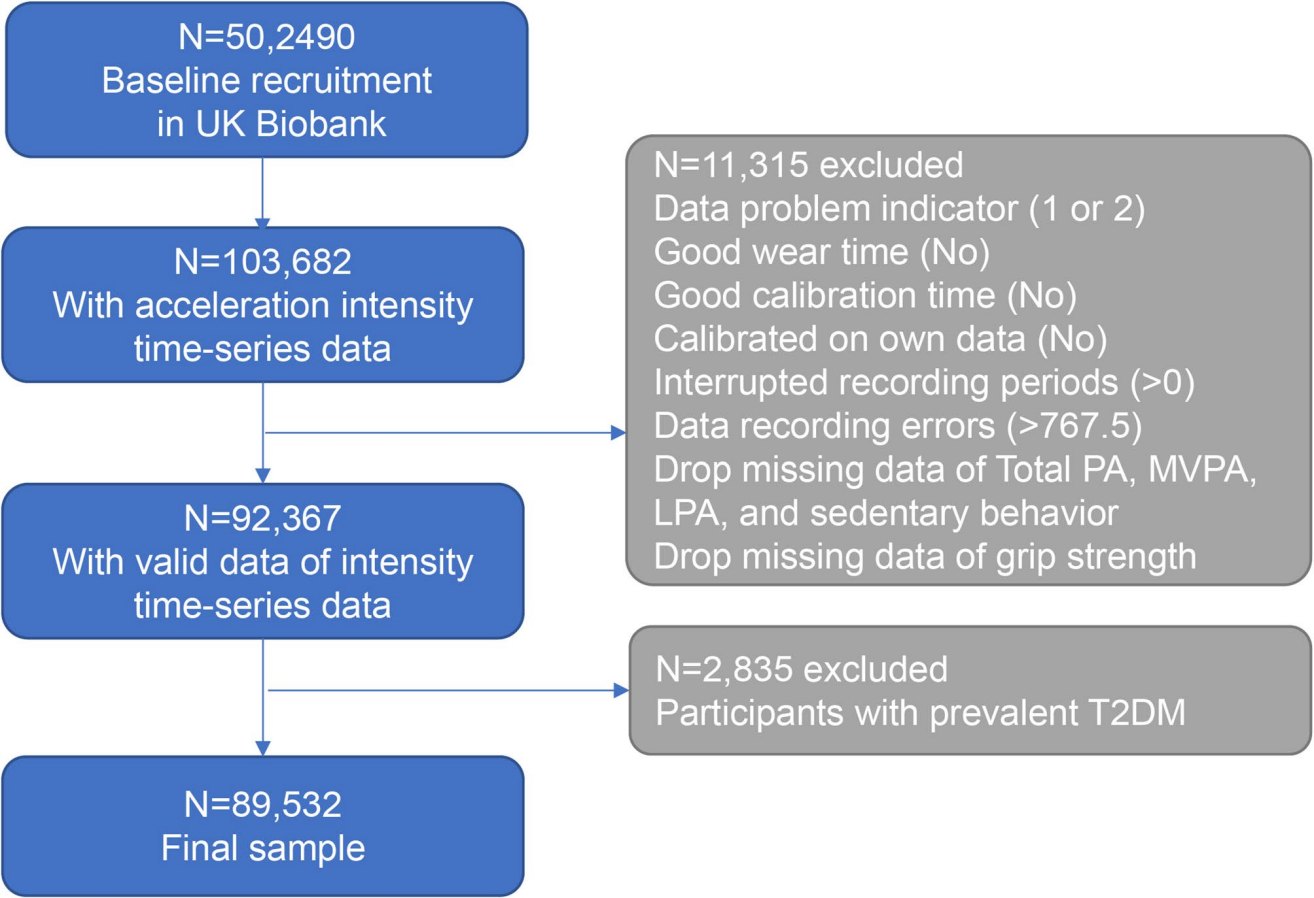
Flow chart of participants enrollments. PA, physical activity; MVPA, moderate-to-vigorous PA; LPA, light PA; T2DM, type 2 diabetes mellitus.

The study included 89,532 participants (mean age: 62.3 years; 57.0% female; 97.0% of white ethnicity). Among them, 39.7% (*n* = 35,625) met the guideline-recommended level of MVPA (≥ 150 min per week). Detailed inclusion and exclusion criteria are presented in Fig. 1. Compared to those not meeting the guidelines, participants who achieved the recommended MVPA level were generally younger, more likely to have higher educational attainment, less likely to be current smokers or alcohol users, and more likely to have better control of body weight, blood lipids, and blood pressure, along with fewer major comorbidities (*P* < 0.05). Similar trends were observed across tertiles of total physical activity volume (Table 1).

**Table 1:** Baseline characteristics of the study participants stratified by levels of MVPA (*N* = 87,487).

Characteristics	Overall (*N* = 89,532)	MVPA (minutes/week)		*P* value
		Not recommended (*N* = 53,907)	Recommended (*N* = 35,625)	
Age at accelerometry (years) [mean (SD)]	62.25 (7.85)	63.46 (7.71)	60.42 (7.71)	< 0.001
Female [n (%)]	50,937 (56.9)	32,584 (60.4)	18,353 (51.5)	< 0.001
White ethnicity [n (%)]	86,849 (97.0)	52,301 (97.0)	34,548 (97.0)	0.721
Townsend deprivation index (median [IQR]) ^a^	– 2.46 [– 3.83, – 0.22]	– 2.50 [– 3.83, – 0.29]	– 2.40 [– 3.81, – 0.10]	< 0.001
*Recruitment center [n (%)]*				
England	80,311 (89.7)	48,303 (89.6)	32,008 (89.8)	< 0.001
Scotland	3324 (3.7)	2205 (4.1)	1119 (3.1)	
Wales	5897 (6.6)	3399 (6.3)	2498 (7.0)	
*Season of accelerometer wear [n (%)]*				
Spring	20,236 (22.6)	11,751 (21.8)	8485 (23.8)	< 0.001
Summer	23,396 (26.1)	13,841 (25.7)	9555 (26.8)	
Autumn	26,775 (29.9)	16,212 (30.1)	10,563 (29.7)	
Winter	19,125 (21.4)	12,103 (22.5)	7022 (19.7)	
Education level [n (%)]				
Degree or above	39,542 (44.2)	21,747 (40.3)	17,795 (50.0)	< 0.001
Any other qualification	42,702 (47.7)	26,974 (50.0)	15,728 (44.1)	
No qualification	7288 (8.1)	5186 (9.6)	2102 (5.9)	
*Obesity status [n (%)]*				
Underweight or normal weight	36,137 (40.4)	18,115 (33.6)	18,022 (50.6)	< 0.001
Overweight	36,818 (41.1)	22,935 (42.5)	13,883 (39.0)	
Obese	16,577 (18.5)	12,857 (23.9)	3720 (10.4)	
Healthy diet score (median [IQR])	3.00 [2.00, 4.00]	3.00 [2.00, 3.00]	3.00 [2.00, 4.00]	< 0.001
*Smoking status [n (%)]*				
Never	51,839 (57.9)	30,295 (56.2)	21,544 (60.5)	< 0.001
Previous	32,074 (35.8)	19,734 (36.6)	12,340 (34.6)	
Current	5619 (6.3)	3878 (7.2)	1741 (4.9)	
*Alcohol consumption [n (%)]*				
Not current	5230 (5.8)	3502 (6.5)	1728 (4.9)	< 0.001
Two or less times a week	41,242 (46.1)	25,755 (47.8)	15,487 (43.5)	
Three or more times a week	43,060 (48.1)	24,650 (45.7)	18,410 (51.7)	
Grip strength (kg) [mean (SD)]	30.33 (10.60)	29.30 (10.59)	31.88 (10.43)	< 0.001
Sleep duration (hours/day) [mean (SD)]	7.28 (0.89)	7.30 (0.91)	7.24 (0.86)	< 0.001
HbA1c (mmol/mol) [mean (SD)]	35.05 (4.68)	35.41 (4.97)	34.50 (4.16)	< 0.001
HbA1c (%) [mean (SD)]	5.36 (0.43)	5.39 (0.45)	5.31 (0.38)	< 0.001
History of hypertension [n (%)]	23,136 (25.8)	16,226 (30.1)	6910 (19.4)	< 0.001
History of high cholesterol [n (%)]	11,697 (13.1)	8392 (15.6)	3305 (9.3)	< 0.001
History of cancers [n (%)]	12,829 (14.3)	8497 (15.8)	4332 (12.2)	< 0.001
History of coronary heart diseases [n (%)]	3979 (4.4)	2949 (5.5)	1030 (2.9)	< 0.001
Use of blood pressure-lowering medications [n (%)]	16,250 (18.1)	11,831 (21.9)	4419 (12.4)	< 0.001
Use of cholesterol-lowering medications [n (%)]	14,082 (15.7)	10,077 (18.7)	4005 (11.2)	< 0.001

Data are presented as mean (SD), or median [IQR], or n (%).

^a^Townsend deprivation index was calculated based on the preceding national census output areas prior to participants joining UK Biobank.

IQR, Interquartile range; MVPA, moderate-to-vigorous physical activity; SD, standard deviation.

### Associations of PA and sedentary behavior with incident T2DM

**Fig. 2:**
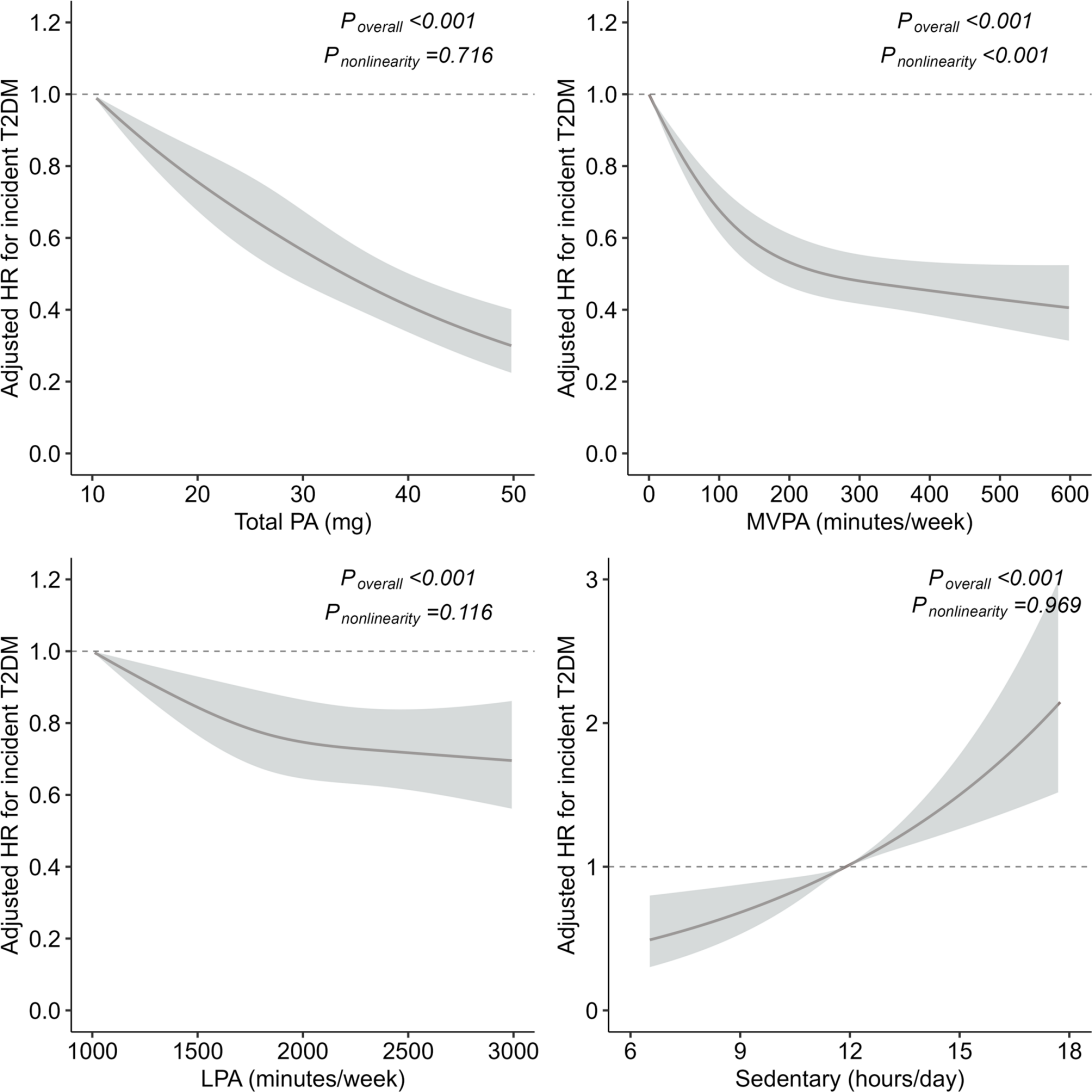
Dose–response associations of the accelerometer-measured total volume of PA, MVPA, LPA, and sedentary behavior with T2DM. The solid line referred to the HRs from the restricted cubic spline regression. Restricted cubic splines were constructed with 3 knots (10th, 50th, 90th). Adjusted HRs (95% CI) were calculated using Cox proportional hazards regression analysis adjusted for age at the time of accelerometer wearing, sex, ethnicity, Townsend deprivation index, recruitment center, education level, the season of accelerometer wearing, obesity status, healthy diet score, sleep duration, grip strength, smoking status, alcohol consumption, HbA1c, hypertension, high cholesterol, history of coronary heart diseases, history of cancers, use of blood pressure-lowering medications, and use of cholesterol-lowering medications. PA, physical activity; MVPA, moderate-to-vigorous PA; LPA, light PA; T2DM, type 2 diabetes mellitus.

Over a median follow-up of 7.0 years (IQR, 6.4–7.5), a total of 2353 incident T2DM cases were documented, corresponding to an overall incidence rate of 3.8 per 1000 person-years. As shown in Fig. 2, the associations of total PA and LPA with incident T2DM were linear (*P* for linearity < 0.001; *P* for nonlinearity > 0.05). In contrast, MVPA showed an inverse and nonlinear dose-response association with T2DM risk (*P* for linearity < 0.001; *P* for nonlinearity < 0.001). Conversely, sedentary time was positively associated with T2DM risk in a linear fashion (*P* for linearity < 0.001; *P* for nonlinearity = 0.969).

In categorical analyses (Table 2), compared with participants in the low total PA group, those with moderate and high volumes had a 20% (HR, 0.80; 95% CI, 0.73–0.88) and 36% (HR, 0.64; 95% CI, 0.56–0.72) lower risk of incident T2DM, respectively. Similarly, participants who met the recommended MVPA level had a 32% lower risk of developing T2DM compared with those who did not (HR, 0.68; 95% CI, 0.61–0.76). A high volume of LPA was also significantly associated with reduced T2DM risk compared to a low volume (HR, 0.88; 95% CI, 0.79–0.98). In contrast, sedentary behavior was associated with an increased risk of incident T2DM (HR, 1.34; 95% CI, 1.19–1.52).

**Table 2:** Associations of the accelerometer-measured total volume of PA, MVPA, LPA, sedentary behavior and sarcopenia status with incident T2DM.

Exposures	*N*	Case/person-years	Incident rate (per 1000 person-years)	Model 1^*^	Model 2^†^	Model 3^‡^
				HR (95% CI)	HR (95% CI)	HR (95% CI)
*Total PA*						
Low	29,868	1320/200,639	6.58	1 (Reference)	1 (Reference)	1 (Reference)
Moderate	29,837	654/205,676	3.18	0.54 (0.49–0.60)	0.68 (0.61–0.74)	0.80 (0.73–0.88)
High	29,827	379/207,698	1.82	0.34 (0.30–0.38)	0.50 (0.44–0.56)	0.64 (0.56–0.72)
*MVPA*						
Not recommended	53,907	1875/366,411	5.12	1 (Reference)	1 (Reference)	1 (Reference)
Recommended	35,625	478/247,602	1.93	0.39 (0.35–0.43)	0.57 (0.52–0.64)	0.68 (0.61–0.76)
*LPA*						
Low	29,844	1035/202,606	5.11	1 (Reference)	1 (Reference)	1 (Reference)
Moderate	29,844	686/205,485	3.34	0.72 (0.66–0.80)	0.81 (0.74–0.90)	0.89 (0.80–0.98)
High	29,844	632/205,922	3.07	0.71 (0.64–0.78)	0.80 (0.72–0.89)	0.88 (0.79–0.98)
*Sedentary behavior*						
Low	29,844	464/206,640	2.25	1 (Reference)	1 (Reference)	1 (Reference)
Moderate	29,844	680/205,429	3.31	1.38 (1.23–1.55)	1.15 (1.02–1.30)	1.11 (0.98–1.25)
High	29,844	1209/201,944	5.99	2.29 (2.06–2.56)	1.48 (1.31–1.66)	1.34 (1.19–1.52)
*Grip strength*						
Normal	82,364	2089/565,760	3.69	1 (Reference)	1 (Reference)	1 (Reference)
Low	7168	264/48,253	5.47	1.36 (1.20–1.55)	1.12 (0.98–1.28)	1.07 (0.94–1.21)
*Walk pace*						
Brisk	4135	627/288,072	2.18	1 (Reference)	1 (Reference)	1 (Reference)
Steady average	43,789	1411/298,863	4.72	2.04 (1.85–2.24)	1.38 (1.25–1.52)	1.25 (1.13–1.38)
Slow	41,608	315/27,077	11.63	4.96 (4.33–5.69)	2.04 (1.77–2.36)	1.82 (1.57–2.10)
*Muscle mass*						
Normal	47,984	934/331,663	2.82	1 (Reference)	1 (Reference)	1 (Reference)
Mild to moderate deficit	35,887	1100/244,687	4.50	1.27 (1.16–1.39)	1.17 (1.07–1.28)	1.22 (1.12–1.34)
Severe deficit	5661	319/37,663	8.47	1.84 (1.61–2.10)	1.22 (1.07–1.40)	1.15 (1.00-1.32)

*Model 1 was adjusted for age at the time of accelerometer wearing and sex

†Model 2 was adjusted for age at the time of accelerometer wearing, sex, ethnicity, Townsend deprivation index, recruitment center, education level, the season of accelerometer wearing, obesity status, healthy diet score, sleep duration, grip strength, smoking status, alcohol consumption, and HbA1c

‡Model 3 was adjusted for age at the time of accelerometer wearing, sex, ethnicity, Townsend deprivation index, recruitment center, education level, the season of accelerometer wearing, obesity status, healthy diet score, sleep duration, grip strength, smoking status, alcohol consumption, HbA1c, hypertension, high cholesterol, history of coronary heart diseases, history of cancers, use of blood pressure-lowering medications, and use of cholesterol-lowering medications

CI, confidence interval; HR, hazard ratio; LPA, light physical activity; MVPA, moderate-to-vigorous physical activity; T2DM, type 2 diabetes mellitus

### Associations between sarcopenia status and incident T2DM

In terms of sarcopenia status, after adjusted for age and sex, compared to participants with normal hand grip strength, those with low grip strength had approximately 36% increased risk of incident T2DM (model 1, HR, 1.36; 95% CI, 1.20–1.55). However, this association was not significant in the fully adjusted model (model 3, HR, 1.07; 95% CI, 0.94–1.21). Steady average and slow walk pace were associated with 25% (HR, 1.25; 95% CI, 1.13–1.38) and 82% (HR, 1.82; 95% CI, 1.57–2.10) increased risk of T2DM compared with a brisk walk pace, respectively. Moreover, muscle mass deficit was associated with higher risk of T2DM (mild to moderate deficit, HR, 1.22, 95% CI, 1.12–1.34; severe deficit, HR, 1.15; 95% CI, 1.00–1.32).

### Associations of PA and sedentary behavior with T2DM stratified by sarcopenia status

**Fig. 3:**
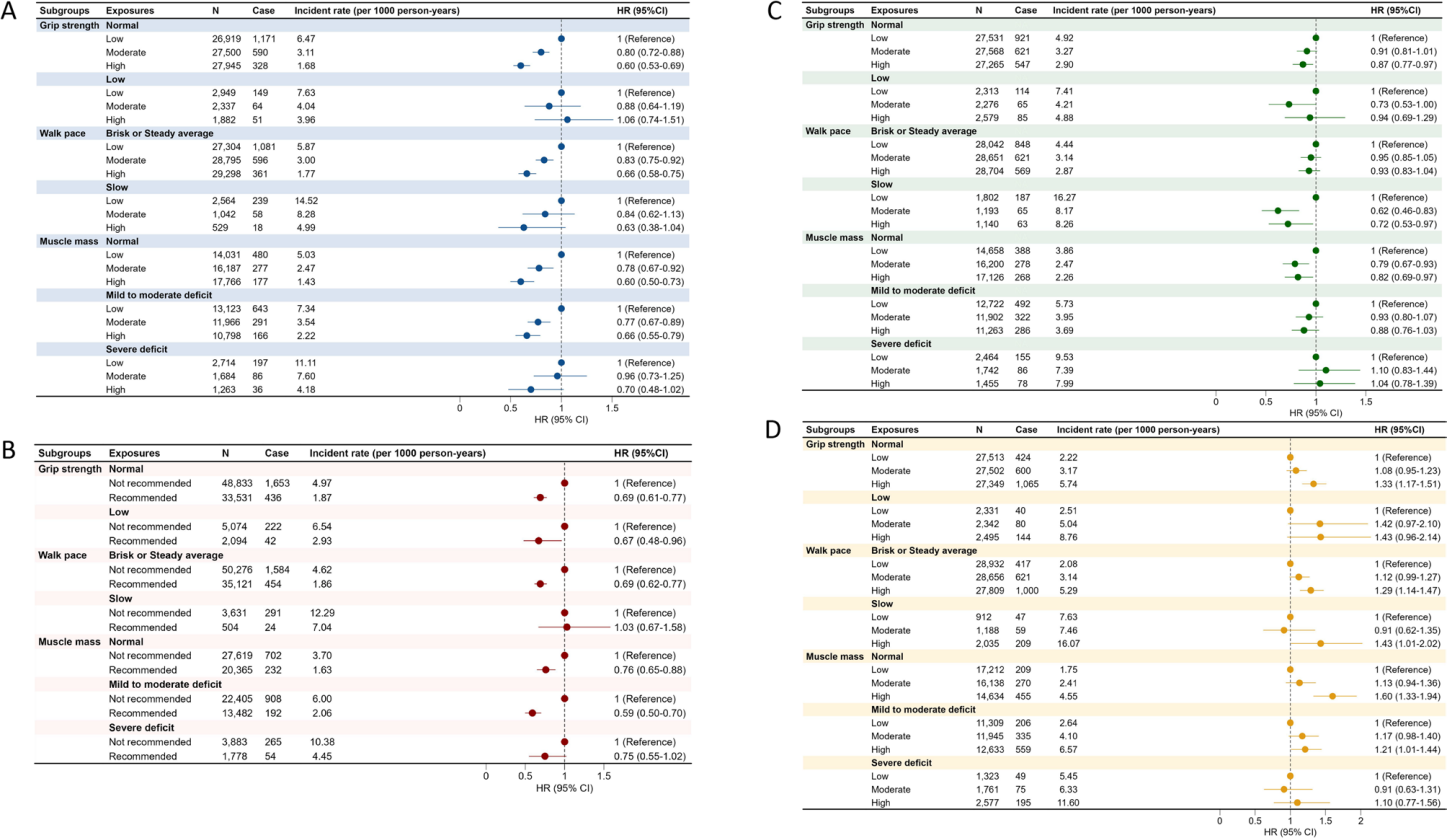
Joint associations of physical activity, sedentary behavior, and grip strength with incident type 2 diabetes, stratified by age and sex. Forest plots display the adjusted hazard ratios (HR) and 95% confidence intervals (CIs) for incident T2DM across subgroups defined by age (< 65 and ≥ 65 years) and sex for individuals with normal versus low grip strength. (**A**) Total volume of physical activity (Total PA); (**B**) Moderate-to-vigorous physical activity (MVPA); (**C**) Light physical activity (LPA); (**D**) Sedentary behavior. Adjusted HRs were calculated using Cox proportional hazards regression models with full adjustments for age, sex, ethnicity, Townsend deprivation index, recruitment center, education level, season of accelerometer wearing, obesity status, healthy diet score, sleep duration, grip strength, smoking status, alcohol consumption, HbA1c, hypertension, high cholesterol, history of coronary heart diseases, history of cancers, and use of blood pressure/cholesterol-lowering medications.

When stratified by levels of grip strength, the risk for T2DM associated with total PA was more pronounced among participants with normal grip strength (high vs. low: HR, 0.60; 95%CI, 0.53–0.69) in contrast with low grip strength (high vs. low: HR, 1.06; 95%CI, 0.74–1.51) (Tables 3, 4, 5). Also, the inverse association between total PA and T2DM risk was more evident among individuals with brisk or steady average walk pace (high vs. low: HR, 0.66; 95% CI, 0.58–0.75), while no significant association was observed in those with low grip strength (HR, 0.63; 95% CI, 0.38–1.04). Furthermore, among participants with normal (high vs. low: HR, 0.60; 95% CI, 0.50–0.73) or mild to moderate (high vs. low: HR, 0.66; 95% CI, 0.55–0.79) deficit muscle mass, higher total PA was associated with a greater reduction in T2DM risk than among those with severe muscle mass deficit (high vs. low: HR, 0.70; 95% CI, 0.48–1.02).Similar patterns were observed among the combinations of sarcopenia status with MVPA, LPA, and sedentary behavior. To further explore these interactions, we conducted deeper subgroup analyses stratified by demographic factors. As illustrated in Fig. 3, the modifying effect of grip strength on the associations of physical activity (Total PA, MVPA, LPA) and sedentary behavior with incident T2DM remained largely consistent across different age (< 65 and ≥ 65 years) and sex subgroups.

**Table 3:** Associations of physical activity and sedentary behavior with incident T2DM stratified by Grip strength.

Subgroups	Exposures	N	Case/person-years	Incident rate (per 1000 person-years)	HR (95% CI)
Grip strength	*Normal*				
	*Total PA*				
	Low	26,919	1171/181,098	6.47	1 (Reference)
	Moderate	27,500	590/189,847	3.11	0.80 (0.72–0.88)
	High	27,945	328/194,815	1.68	0.60 (0.53–0.69)
	*MVPA*				
	Not recommended	48,833	1653/332,491	4.97	1 (Reference)
	Recommended	33,531	436/233,269	1.87	0.69 (0.61–0.77)
	*LPA*				
	Low	27,531	921/187,216	4.92	1 (Reference)
	Moderate	27,568	621/190,055	3.27	0.91 (0.81–1.01)
	High	27,265	547/188,489	2.9	0.87 (0.77–0.97)
	*Sedentary behavior*				
	Low	27,513	424/190,682	2.22	1 (Reference)
	Moderate	27,502	600/189,570	3.17	1.08 (0.95–1.23)
	High	27,349	1065/185,507	5.74	1.33 (1.17–1.51)
	*Low*				
	*Total PA*				
	Low	2949	149/19,540	7.63	1 (Reference)
	Moderate	2337	64/15,829	4.04	0.88 (0.64–1.19)
	High	1882	51/12,883	3.96	1.06 (0.74–1.51)
	*MVPA*				
	Not recommended	5074	222/33,920	6.54	1 (Reference)
	Recommended	2094	42/14,332	2.93	0.67 (0.48–0.96)
	*LPA*				
	Low	2313	114/15,391	7.41	1 (Reference)
	Moderate	2276	65/15,429	4.21	0.73 (0.53–1.00)
	High	2579	85/17,433	4.88	0.94 (0.69–1.29)
	*Sedentary behavior*				
	Low	2331	40/15,957	2.51	1 (Reference)
	Moderate	2342	80/15,858	5.04	1.42 (0.97–2.10)
	High	2495	144/16,437	8.76	1.43 (0.96–2.14)

**Table 4:** Associations of physical activity and sedentary behavior with incident T2DM stratified by Walk pace.

Subgroups	Exposures	N	Case/person-years	Incident rate (per 1000 person-years)	HR (95% CI)
Walk pace	*Brisk or Steady average*				
	*Total PA*				
	Low	27,304	1081/184,179	5.87	1 (Reference)
	Moderate	28,795	596/198,667	3	0.83 (0.75–0.92)
	High	29,298	361/204,089	1.77	0.66 (0.58–0.75)
	*MVPA*				
	Not recommended	50,276	1584/342,741	4.62	1 (Reference)
	Recommended	35,121	454/244,195	1.86	0.69 (0.62–0.77)
	*LPA*				
	Low	28,042	848/191,113	4.44	1 (Reference)
	Moderate	28,651	621/197,526	3.14	0.95 (0.85–1.05)
	High	28,704	569/198,296	2.87	0.93 (0.83–1.04)
	*Sedentary behavior*				
	Low	28,932	417/200,476	2.08	1 (Reference)
	Moderate	28,656	621/197,521	3.14	1.12 (0.99–1.27)
	High	27,809	1000/188,939	5.29	1.29 (1.14–1.47)
	*Slow*				
	*Total PA*				
	Low	2564	239/16,459	14.52	1 (Reference)
	Moderate	1042	58/7009	8.28	0.84 (0.62–1.13)
	High	529	18/3609	4.99	0.63 (0.38–1.04)
	*MVPA*				
	Not recommended	3631	291/23,670	12.29	1 (Reference)
	Recommended	504	24/3407	7.04	1.03 (0.67–1.58)
	*LPA*				
	Low	1802	187/11,494	16.27	1 (Reference)
	Moderate	1193	65/7958	8.17	0.62 (0.46–0.83)
	High	1140	63/7625	8.26	0.72 (0.53–0.97)
	*Sedentary behavior*				
	Low	912	47/6164	7.63	1 (Reference)
	Moderate	1188	59/7908	7.46	0.91 (0.62–1.35)
	High	2035	209/13,005	16.07	1.43 (1.01–2.02)

**Table 5:** Associations of physical activity and sedentary behavior with incident T2DM stratified by Muscle mass deficit.

Subgroups	Exposures	N	Case/person-years	Incident rate (per 1000 person-years)	HR (95% CI)
Muscle mass	*Normal*				
	*Total PA*				
	Low	14,031	480/95,337	5.03	1 (Reference)
	Moderate	16,187	277/112,164	2.47	0.78 (0.67–0.92)
	High	17,766	177/124,162	1.43	0.60 (0.50–0.73)
	*MVPA*				
	Not recommended	27,619	702/189,619	3.7	1 (Reference)
	Recommended	20,365	232/142,044	1.63	0.76 (0.65–0.88)
	*LPA*				
	Low	14,658	388/100,548	3.86	1 (Reference)
	Moderate	16,200	278/112,372	2.47	0.79 (0.67–0.93)
	High	17,126	268/118,743	2.26	0.82 (0.69–0.97)
	*Sedentary behavior*				
	Low	17,212	209/119,609	1.75	1 (Reference)
	Moderate	16,138	270/111,944	2.41	1.13 (0.94–1.36)
	High	14,634	455/100,110	4.55	1.60 (1.33–1.94)
	*Mild to moderate deficit*				
	*Total PA*				
	Low	13,123	643/87,566	7.34	1 (Reference)
	Moderate	11,966	291/82,199	3.54	0.77 (0.67–0.89)
	High	10,798	166/74,922	2.22	0.66 (0.55–0.79)
	*MVPA*				
	Not recommended	22,405	908/151,261	6	1 (Reference)
	Recommended	13,482	192/93,426	2.06	0.59 (0.50–0.70)
	*LPA*				
	Low	12,722	492/85,796	5.73	1 (Reference)
	Moderate	11,902	322/81,473	3.95	0.93 (0.80–1.07)
	High	11,263	286/77,418	3.69	0.88 (0.76–1.03)
	*Sedentary behavior*				
	Low	11,309	206/78,033	2.64	1 (Reference)
	Moderate	11,945	335/81,633	4.1	1.17 (0.98–1.40)
	High	12,633	559/85,021	6.57	1.21 (1.01–1.44)
	*Severe deficit*				
	*Total PA*				
	Low	2714	197/17,736	11.11	1 (Reference)
	Moderate	1684	86/11,313	7.6	0.96 (0.73–1.25)
	High	1263	36/8615	4.18	0.70 (0.48–1.02)
	*MVPA*				
	Not recommended	3883	265/25,532	10.38	1 (Reference)
	Recommended	1778	54/12,131	4.45	0.75 (0.55–1.02)
	*LPA*				
	Low	2464	155/16,262	9.53	1 (Reference)
	Moderate	1742	86/11,639	7.39	1.10 (0.83–1.44)
	High	1455	78/9761	7.99	1.04 (0.78–1.39)
	*Sedentary behavior*				
	Low	1323	49/8997	5.45	1 (Reference)
	Moderate	1761	75/11,852	6.33	0.91 (0.63–1.31)
	High	2577	195/16,814	11.6	1.10 (0.77–1.56)

HR was adjusted for age at the time of accelerometer wearing, sex, ethnicity, Townsend deprivation index, recruitment center, education level, the season of accelerometer wearing, obesity status, healthy diet score, sleep duration, grip strength, smoking status, alcohol consumption, HbA1c, hypertension, high cholesterol, history of coronary heart diseases, history of cancers, use of blood pressure-lowering medications, and use of cholesterol-lowering medications. CI, confidence interval; HR, hazard ratio; LPA, light physical activity; MVPA, moderate-to-vigorous physical activity; T2DM, type 2 diabetes mellitus.

### Sensitivity and subgroup analyses

In sensitivity analyses, the observed associations of total PA, sedentary behavior, and sarcopenia status with new-onset T2DM remained largely robust when analyses were restricted to participants with complete data (Supplementary Figure S1 and Supplementary Table S3), when early T2DM events (within the first 2 years of follow-up) were excluded (Supplementary Figure S2 and Supplementary Table S4) and after accounting for the competing risk of death (Supplementary Table S5).The associations of PA, sedentary behavior, and sarcopenia status with the risk of incident T2DM were broadly consistent across subgroups defined by age (< 65 years vs. ≥ 65 years), sex (female vs. male), and obesity status (underweight or normal vs. overweight) (Supplementary Table S6–S8).

## Discussion

In this large-scale accelerometry-based prospective cohort study, we found a dose-response relationship between sedentary behavior, LPA, MVPA and the risk of T2DM. Higher levels of PA and lower sedentary time are independently associated with a reduced risk of incident T2DM. Secondly, we found that sarcopenia status was independently associated with the risk of incident T2DM. Finally, grip strength significantly modified the association between PA and T2DM. Future policies and guidelines should encourage small, achievable increases in PA, targeted interventions for those with low grip strength to reduce the risk of incident T2DM.

Notably, we extend prior research by emphasizing that even modest improvements below current guidelines provide clinically meaningful benefits^37,38^. We quantified the risk gradients across PA and sedentary behavior categories. Compared with those in the lowest tertile, participants in the highest tertile of total PA had a 36% lower risk of developing T2DM after full multivariable adjustment. Notably, achieving guideline-recommended levels of MVPA was associated with a substantial reduction in T2DM incidence, while high sedentary behavior was associated with a 34% increased risk, even after considering PA and other covariates. The protective association of LPA, though significant, was less pronounced than that of MVPA, underscoring the superior metabolic benefit of higher intensity activity for diabetes prevention^39^. Consistent with recent findings demonstrating muscle strength’s independent protective effects irrespective of genetic predisposition, our results highlight that higher muscle strength significantly reduces diabetes risk even after accounting for PA, sedentary behavior, and numerous traditional confounders^17^.

Unlike previous studies that predominantly focused on muscle mass or genetic susceptibility alone, our findings also provide novel insights regarding the interplay between PA, sedentary behavior, and markers of grip strength and mass^23^. The inverse association between PA and T2DM risk was more prominent among individuals with preserved grip strength or muscle mass, whereas these associations were attenuated and sometimes non-significant in those with muscle deficits. This suggests that preserved muscle function may enhance the glycemic benefit of physical activity, potentially through improved glucose uptake and insulin sensitivity in skeletal muscle^40,41^. Conversely, low grip strength and muscle mass may reflect underlying sarcopenia, which could reduce the effectiveness of PA in diabetes prevention, and might itself be an early marker of metabolic vulnerability^42,43^. Furthermore, our results support the notion of a dose–response relationship, with incremental reductions in T2DM risk observed across increasing tertiles of PA, as well as with increased MVPA duration^44,45^. Importantly, even modest increases in PA, well below current guideline thresholds, were associated with meaningful reductions in risk, providing a practical and inclusive public health message. Our stratified analyses by muscle status, walking pace, and other clinical subgroups highlight the potential value of tailoring PA recommendations based on baseline physical capacity and function, rather than applying uniform targets. Potential mechanisms underpinning these associations may include improvements in body composition, insulin action, systemic inflammation, and vascular health mediated by regular physical activity. In contrast, excessive sedentary time may promote ectopic fat deposition, impair insulin signaling, and exacerbate chronic low-grade inflammation^46,47^.

Furthermore, our findings suggest that functional grip strength assessments, such as grip strength, are more relevant metabolic predictors compared to muscle mass measurements alone, aligning with recent recommendations for muscle quality over quantity evaluation^48^. While our findings align with and extend previous accelerometer-based studies, they underscore the clinical relevance of both promoting movement and targeting muscular health as components of comprehensive diabetes prevention strategies^13^.

### Clinical Implications

Our study provides actionable evidence supporting the integration of device-measured PA and muscle function assessment into risk stratification and T2DM prevention efforts^48^. The clear risk gradient observed across PA and sedentary behavior categories emphasizes the need for pragmatic strategies that encourage regular movement and minimize prolonged sedentary behavior in daily life not only in high-risk groups but across the general adult population^49^. While achieving recommended MVPA levels should remain an aspirational goal, even incremental increases in habitual activity, or shifting from low to moderate activity, confer substantial metabolic benefits.

The attenuation of PA’s protective association in participants with low grip strength or reduced muscle mass suggests that early identification and intervention for sarcopenia should be considered in diabetes prevention programs. Handgrip strength is a feasible and reproducible measure in clinical settings and could help individualize PA recommendations. Clinically, our findings advocate for structured multidimensional interventions combining aerobic exercise, resistance training, and functional exercises to optimize metabolic health and diabetes prevention, particularly among older adults or those with reduced physical function.

### Limitations

Several limitations should be acknowledged. Similar to most observational studies, the associations we report cannot definitively establish causality, although they are robustly adjusted for numerous confounders. Our study measured physical activity and grip strength at baseline only, potentially missing changes over time, which might slightly underestimate their true associations with diabetes risk. Additionally, our cohort primarily consisted of White British individuals, limiting the generalizability of findings to ethnically diverse populations. Lastly, we did not capture the timing of activity within the day, although emerging evidence indicates that circadian alignment could influence metabolic responses to physical activity, especially in shift workers.

## Conclusion

In conclusion, our study integrated objective measurements of PA and sedentary behavior, demonstrating the dose-response association between higher levels of PA and decreased incident T2DM risk. Moreover, sarcopenia status was an independent risk factor for T2DM. Grip strength significantly modified the association between PA and incident T2DM risk. These findings highlight the importance of addressing both movement behaviors and muscle function in T2DM prevention. Future policies should adopt integrated interventions that target both PA and muscular health to more effectively reduce the burden of T2DM.

## Supplementary Information

Below is the link to the electronic supplementary material.

Supplementary Material 1

## Data Availability

The datasets used and/or analysed during the current study available from the corresponding author on reasonable request.

## Abbreviations

ALST: Appendicular lean soft tissue
BIA: Bioelectrical impedance analysis
BMI: Body mass index
CI: Confidence interval
EWGSOP2: European Working Group on Sarcopenia in Older People 2
FFM: Fat-free mass
HbA1c: Glycated haemoglobin A1c
HR: Hazard ratio
IQR: Interquartile range
LPA: Light-intensity physical activity
MVPA: Moderate-to-vigorous physical activity
PA: Physical activity
T2DM: Type 2 diabetes mellitus
VPA: Vigorous physical activity
