# Supplementary material for: Accelerometer-derived physical activity, sarcopenia, and grip strength as modifiers of type 2 diabetes risk
Source: Sci Rep. 2026 May 28;16:24470. doi: 10.1038/s41598-026-54864-8 (PMC13451494; doi:10.1038/s41598-026-54864-8)
Supplement: Supplementary file 1 [file 41598_2026_54864_MOESM1_ESM.docx]

**Supplementary Material**

**Title:**

| Supplementary Table S1 | Information about exposures, outcomes, and covariates | 2 |
| --- | --- | --- |
| Supplementary Table S2 | The number (percentage) of participants with missing covariate data in the analytic sample | 3 |
| Supplementary Figure S1 | Sensitivity analyses of dose-response associations of total volume of PA, MVPA, LPA, and sedentary behavior with incident T2DM after excluding participants with missing data on covariables | 4 |
| Supplementary Table S3 | Sensitivity analyses of associations of total volume of PA, MVPA, LPA, and sedentary behavior with incident T2DM after excluding participants with missing data on covariables | 5 |
| Supplementary Figure S2 | Sensitivity analyses of dose-resonse associations of total volume of PA, MVPA, LPA, and sedentary behavior with incident T2DM after excluding events occurring within the first two years | 7 |
| Supplementary Table S4 | Sensitivity analyses of associations of total volume of PA, MVPA, LPA, and sedentary behavior with incident T2DM after excluding events occurring within the first two years | 8 |
| Supplementary Table S5 | Sensitive analyses of associations of total volume of PA, MVPA, LPA, and sedentary behavior with incident T2DM by using competing risk regression | 10 |
| Supplementary Table S6 | Subgroup analyses of associations of total volume of PA, MVPA, LPA, and sedentary behavior with incident T2DM stratified by age | 12 |
| Supplementary Table S7 | Subgroup analyses of associations of total volume of PA, MVPA, LPA, and sedentary behavior with incident T2DM stratified by sex | 15 |
| Supplementary Table S8 | Subgroup analyses of associations of total volume of PA, MVPA, LPA, and sedentary behavior with incident T2DM stratified by obese status | 18 |

**Supplementary Table S1. Information about exposures, outcomes, and covariates**

| **Variables** | **Field ID** | **ICD-10 code** |
| --- | --- | --- |
| T2DM |  | E11 |
| PA and sedentary | 90001 |  |
| Grip strength | 46/47 |  |
| Muscle mass | 21001/23101/23113/23117/23121/23125 |  |
| Walk pace | 924 |  |
| Age at accelerometry | 21003 |  |
| Sex | 31 |  |
| Ethnicity | 21000 |  |
| Townsend deprivation index | 189 |  |
| Recruitment center | 54 |  |
| Season of accelerometer wear | 90010 |  |
| Education level | 6138 |  |
| Obesity status | 21001 |  |
| Healthy diet score | 1289/1299/1309/1319/1329/1339/1349/1369/1379/1389 |  |
| Smoking status | 20116 |  |
| Alcohol consumption | 1558 |  |
| Sleep duration | 90001 |  |
| HbA1c | 30750 |  |
| History of hypertension | 6150 | I10 |
| History of high cholesterol | 20002 | E78 |
| History of coronary heart diseases |  | I20-25 |
| History of cancers | 2453 | C00-C97 |
| Use of cholesterol-lowering medications | 20003 |  |
| Use of blood pressure-lowering medications | 20003/6153/6177 |  |

ICD-10, International Classification of Diseases; T2DM, type 2 diabetes mellitus.

**Supplementary Table S2. The number (percentage) of participants with missing covariate data in the analytic sample.**

| **Variables** | **N** | **%** |
| --- | --- | --- |
| Age at accelerometry | 0 | 0.00 |
| Sex | 0 | 0.00 |
| Ethnicity | 301 | 0.34 |
| Townsend deprivation index | 103 | 0.12 |
| Recruitment center | 0 | 0.00 |
| Season of accelerometer wear | 0 | 0.00 |
| Education level | 492 | 0.55 |
| Obesity status | 77 | 0.09 |
| Grip strength | 0 | 0.00 |
| Healthy diet score | 32 | 0.04 |
| Smoking status | 192 | 0.21 |
| Alcohol consumption | 51 | 0.06 |
| Sleep duration | 0 | 0.00 |
| HbA1c | 5221 | 5.83 |
| History of hypertension | 88 | 0.10 |
| History of high cholesterol | 0 | 0.00 |
| History of coronary heart diseases | 0 | 0.00 |
| History of cancers | 150 | 0.17 |
| Use of cholesterol-lowering medications | 289 | 0.32 |
| Use of blood pressure-lowering medications | 424 | 0.47 |

**Supplementary Figure S1. Sensitivity analyses of dose-response associations of total volume of PA, MVPA, LPA, and sedentary behavior with incident T2DM after excluding participants with missing data on covariables.**

PA, physical activity; MVPA, moderate-to-vigrious PA; LPA, light PA; T2DM, type 2 diabetes mellitus.

**Supplementary Table S3. Sensitivity analyses of associations of total volume of PA, MVPA, LPA, and sedentary behavior with incident T2DM after excluding participants with missing data on covariables**

| **Exposures** | **N** | **Case/person-years** | **Incident rate**  **(per 1000 person-years)** | **Model 1^*^** | **Model 2^†^** | **Model 3^‡^** |
| --- | --- | --- | --- | --- | --- | --- |
|  |  |  |  | **HR (95% CI)** | **HR (95% CI)** | **HR (95% CI)** |
| **Total PA** |  |  |  |  |  |  |
| Low | 27,417 | 1183/184312 | 6.42 | 1 (Reference) | 1 (Reference) | 1 (Reference) |
| Moderate | 27,342 | 589/188522 | 3.12 | 0.55 (0.50-0.61) | 0.68 (0.61-0.75) | 0.80 (0.72-0.89) |
| High | 27,375 | 345/190628 | 1.81 | 0.35 (0.31-0.39) | 0.51 (0.45-0.58) | 0.66 (0.58-0.75) |
| **MVPA** |  |  |  |  |  |  |
| Not recommended | 49,303 | 1683/335278 | 5.02 | 1 (Reference) | 1 (Reference) | 1 (Reference) |
| Recommended | 32,831 | 434/228184 | 1.9 | 0.39 (0.35-0.44) | 0.58 (0.52-0.64) | 0.70 (0.63-0.78) |
| **LPA** |  |  |  |  |  |  |
| Low | 27,378 | 916/185982 | 4.93 | 1 (Reference) | 1 (Reference) | 1 (Reference) |
| Moderate | 27,380 | 635/188527 | 3.37 | 0.76 (0.69-0.84) | 0.85 (0.77-0.94) | 0.90 (0.81-1.00) |
| High | 27,376 | 566/188953 | 3 | 0.72 (0.65-0.80) | 0.81 (0.72-0.91) | 0.89 (0.80-1.00) |
| **Sedentary behavior** |  |  |  |  |  |  |
| Low | 27,378 | 422/189604 | 2.23 | 1 (Reference) | 1 (Reference) | 1 (Reference) |
| Moderate | 27,378 | 617/188418 | 3.27 | 1.38 (1.22-1.56) | 1.14 (1.01-1.30) | 1.10 (0.97-1.25) |
| High | 27,378 | 1078/185440 | 5.81 | 2.25 (2.00-2.52) | 1.44 (1.27-1.63) | 1.30 (1.15-1.48) |
| **Grip strength** |  |  |  |  |  |  |
| Normal | 75,619 | 1884/519577 | 3.63 | 1 (Reference) | 1 (Reference) | 1 (Reference) |
| Low | 6,515 | 233/43884 | 5.31 | 1.34 (1.17-1.54) | 1.11 (0.97-1.28) | 1.03 (0.90-1.19) |
| **Walk pace** |  |  |  |  |  |  |
| Brisk | 38,417 | 273/24046 | 11.35 | 1 (Reference) | 1 (Reference) | 1 (Reference) |
| Steady average | 40,054 | 1271/273468 | 4.65 | 2.03 (1.84-2.24) | 1.37 (1.24-1.52) | 1.24 (1.12-1.38) |
| Slow | 3,663 | 573/265948 | 2.15 | 4.89 (4.23-5.66) | 2.01 (1.72-2.34) | 1.74 (1.49-2.03) |
| **Musle mass** |  |  |  |  |  |  |
| Normal | 44,075 | 855/304599 | 2.81 | 1 (Reference) | 1 (Reference) | 1 (Reference) |
| Mild to moderate deficit | 32,974 | 976/224976 | 4.34 | 1.23 (1.12-1.35) | 1.13 (1.03-1.25) | 1.18 (1.07-1.30) |
| Severe deficit | 5,085 | 286/33886 | 8.44 | 1.83 (1.59-2.11) | 1.23 (1.06-1.41) | 1.14 (0.99-1.32) |

*Model 1 was adjusted for age at the time of accelerometer wearing and sex.

†Model 2 was adjusted for age at the time of accelerometer wearing, sex, ethnicity, Townsend deprivation index, recruitment center, education level, the season of accelerometer wearing, obesity status, healthy diet score, sleep duration, grip strength, smoking status, alcohol consumption, and HbA1c.

‡Model 3 was adjusted for age at the time of accelerometer wearing, sex, ethnicity, Townsend deprivation index, recruitment center, education level, the season of accelerometer wearing, obesity status, healthy diet score, sleep duration, grip strength, smoking status, alcohol consumption, HbA1c, hypertension, high cholesterol, history of coronary heart diseases, history of cancers, use of blood pressure-lowering medications, and use of cholesterol-lowering medications.

CI, confidence interval; HR, hazard ratio; LPA, light physical activity; MVPA, moderate-to-vigorous physical activity; T2DM, type 2 diabetes mellitus.

**Supplementary FigureS2. Sensitivity analyses of dose-resonse associations of total volume of PA, MVPA, LPA, and sedentary behavior with incident T2DM after excluding events occurring within the first two years.**

PA, physical activity; MVPA, moderate-to-vigrious PA; LPA, light PA; T2DM, type 2 diabetes mellitus.

**Supplementary Table S4. Sensitivity analyses of associations of total volume of PA, MVPA, LPA, and sedentary behavior with incident T2DM after excluding events occurring within the first two years**

| **Exposures** | **N** | **Case/person-years** | **Incident rate**  **(per 1000 person-years)** | **Model 1^*^** | **Model 2^†^** | **Model 3^‡^** |
| --- | --- | --- | --- | --- | --- | --- |
|  |  |  |  | **HR (95% CI)** | **HR (95% CI)** | **HR (95% CI)** |
| **Total PA** |  |  |  |  |  |  |
| Low | 29,479 | 923/201811 | 4.57 | 1 (Reference) | 1 (Reference) | 1 (Reference) |
| Moderate | 29,505 | 469/204952 | 2.29 | 0.56 (0.50-0.62) | 0.69 (0.62-0.78) | 0.81 (0.72-0.91) |
| High | 29,447 | 273/206063 | 1.32 | 0.35 (0.31-0.41) | 0.51 (0.44-0.59) | 0.65 (0.56-0.75) |
| **MVPA** |  |  |  |  |  |  |
| Not recommended | 53,020 | 1318/365458 | 3.61 | 1 (Reference) | 1 (Reference) | 1 (Reference) |
| Recommended | 35,411 | 347/247368 | 1.4 | 0.40 (0.36-0.45) | 0.58 (0.51-0.66) | 0.69 (0.60-0.78) |
| **LPA** |  |  |  |  |  |  |
| Low | 29,477 | 709/203187 | 3.49 | 1 (Reference) | 1 (Reference) | 1 (Reference) |
| Moderate | 29,480 | 489/204763 | 2.39 | 0.75 (0.67-0.85) | 0.85 (0.76-0.96) | 0.91 (0.81-1.02) |
| High | 29,474 | 467/204876 | 2.28 | 0.76 (0.68-0.86) | 0.87 (0.77-0.99) | 0.96 (0.85-1.09) |
| **Sedentary behavior** |  |  |  |  |  |  |
| Low | 29,477 | 343/205262 | 1.67 | 1 (Reference) | 1 (Reference) | 1 (Reference) |
| Moderate | 29,477 | 483/204664 | 2.36 | 1.33 (1.16-1.52) | 1.11 (0.96-1.27) | 1.07 (0.93-1.23) |
| High | 29,477 | 839/202900 | 4.14 | 2.15 (1.89-2.44) | 1.38 (1.20-1.59) | 1.27 (1.10-1.47) |
| **Grip strength** |  |  |  |  |  |  |
| Normal | 81,387 | 1476/564709 | 2.61 | 1 (Reference) | 1 (Reference) | 1 (Reference) |
| Low | 7,044 | 189/48117 | 3.93 | 1.39 (1.19-1.62) | 1.15 (0.98-1.34) | 1.08 (0.93-1.26) |
| **Walk pace** |  |  |  |  |  |  |
| Brisk | 41,297 | 203/26904 | 7.55 | 1 (Reference) | 1 (Reference) | 1 (Reference) |
| Steady average | 43,162 | 1004/298193 | 3.37 | 2.00 (1.79-2.23) | 1.36 (1.21-1.52) | 1.24 (1.10-1.39) |
| Slow | 3,972 | 458/287729 | 1.59 | 4.46 (3.78-5.27) | 1.86 (1.56-2.22) | 1.71 (1.43-2.04) |
| **Musle mass** |  |  |  |  |  |  |
| Normal | 47,573 | 690/331224 | 2.08 | 1 (Reference) | 1 (Reference) | 1 (Reference) |
| Mild to moderate deficit | 35,355 | 764/244108 | 3.13 | 1.20 (1.08-1.34) | 1.11 (1.00-1.24) | 1.17 (1.05-1.30) |
| Severe deficit | 5,503 | 211/37494 | 5.63 | 1.67 (1.42-1.96) | 1.12 (0.95-1.32) | 1.07 (0.91-1.26) |

*Model 1 was adjusted for age at the time of accelerometer wearing and sex.

†Model 2 was adjusted for age at the time of accelerometer wearing, sex, ethnicity, Townsend deprivation index, recruitment center, education level, the season of accelerometer wearing, obesity status, healthy diet score, sleep duration, grip strength, smoking status, alcohol consumption, and HbA1c.

‡Model 3 was adjusted for age at the time of accelerometer wearing, sex, ethnicity, Townsend deprivation index, recruitment center, education level, the season of accelerometer wearing, obesity status, healthy diet score, sleep duration, grip strength, smoking status, alcohol consumption, HbA1c, hypertension, high cholesterol, history of coronary heart diseases, history of cancers, use of blood pressure-lowering medications, and use of cholesterol-lowering medications.

CI, confidence interval; HR, hazard ratio; LPA, light physical activity; MVPA, moderate-to-vigorous physical activity; T2DM, type 2 diabetes mellitus.

**Supplementary Table S5. Sensitive analyses of associations of total volume of PA, MVPA, LPA, and sedentary behavior with incident T2DM by using competing risk regression**

| **Exposures** | **N** | **Case/person-years** | **Incident rate**  **(per 1000 person-years)** | **Model 1^*^** | **Model 2^†^** | **Model 3^‡^** |
| --- | --- | --- | --- | --- | --- | --- |
|  |  |  |  | **HR (95% CI)** | **HR (95% CI)** | **HR (95% CI)** |
| **Total PA** |  |  |  |  |  |  |
| Low | 29,868 | 1320/200639 | 6.58 | 1 (Reference) | 1 (Reference) | 1 (Reference) |
| Moderate | 29,837 | 654/205676 | 3.18 | 0.55 (0.50-0.60) | 0.68 (0.62-0.75) | 0.81 (0.73-0.90) |
| High | 29,827 | 379/207698 | 1.82 | 0.35 (0.31-0.39) | 0.50 (0.44-0.57) | 0.64 (0.56-0.73) |
| **MVPA** |  |  |  |  |  |  |
| Not recommended | 53,907 | 1875/366411 | 5.12 | 1 (Reference) | 1 (Reference) | 1 (Reference) |
| Recommended | 35,625 | 478/247602 | 1.93 | 0.39 (0.36-0.44) | 0.58 (0.52-0.64) | 0.69 (0.62-0.77) |
| **LPA** |  |  |  |  |  |  |
| Low | 29,844 | 1035/202606 | 5.11 | 1 (Reference) | 1 (Reference) | 1 (Reference) |
| Moderate | 29,844 | 686/205485 | 3.34 | 0.73 (0.66-0.80) | 0.82 (0.74-0.90) | 0.89 (0.79-0.99) |
| High | 29,844 | 632/205922 | 3.07 | 0.71 (0.64-0.79) | 0.81 (0.72-0.90) | 0.88 (0.78-0.99) |
| **Sedentary behavior** |  |  |  |  |  |  |
| Low | 29,844 | 464/206640 | 2.25 | 1 (Reference) | 1 (Reference) | 1 (Reference) |
| Moderate | 29,844 | 680/205429 | 3.31 | 1.38 (1.23-1.55) | 1.16 (1.03-1.30) | 1.11 (0.98-1.26) |
| High | 29,844 | 1209/201944 | 5.99 | 2.27 (2.04-2.54) | 1.46 (1.30-1.65) | 1.33 (1.17-1.51) |
| **Grip strength** |  |  |  |  |  |  |
| Normal | 82,364 | 2089/565760 | 3.69 | 1 (Reference) | 1 (Reference) | 1 (Reference) |
| Low | 7,168 | 264/48253 | 5.47 | 1.36 (1.19-1.55) | 1.12 (0.98-1.28) | 1.06 (0.92-1.22) |
| **Walk pace** |  |  |  |  |  |  |
| Brisk | 4,135 | 627/288072 | 2.18 | 1 (Reference) | 1 (Reference) | 1 (Reference) |
| Steady average | 43,789 | 1411/298863 | 4.72 | 2.03 (1.85-2.24) | 1.38 (1.25-1.52) | 1.25 (1.13-1.38) |
| Slow | 41,608 | 315/27077 | 11.63 | 4.87 (4.24-5.59) | 2.01 (1.74-2.33) | 1.79 (1.53-2.10) |
| **Musle mass** |  |  |  |  |  |  |
| Normal | 47,984 | 934/331663 | 2.82 | 1 (Reference) | 1 (Reference) | 1 (Reference) |
| Mild to moderate deficit | 35,887 | 1100/244687 | 4.50 | 1.28 (1.16-1.40) | 1.17 (1.07-1.28) | 1.23 (1.11-1.36) |
| Severe deficit | 5,661 | 319/37663 | 8.47 | 1.84 (1.60-2.11) | 1.22 (1.06-1.40) | 1.16 (0.99-1.36) |

*Model 1 was adjusted for age at the time of accelerometer wearing and sex.

†Model 2 was adjusted for age at the time of accelerometer wearing, sex, ethnicity, Townsend deprivation index, recruitment center, education level, the season of accelerometer wearing, obesity status, healthy diet score, sleep duration, grip strength, smoking status, alcohol consumption, and HbA1c.

‡Model 3 was adjusted for age at the time of accelerometer wearing, sex, ethnicity, Townsend deprivation index, recruitment center, education level, the season of accelerometer wearing, obesity status, healthy diet score, sleep duration, grip strength, smoking status, alcohol consumption, HbA1c, hypertension, high cholesterol, history of coronary heart diseases, history of cancers, use of blood pressure-lowering medications, and use of cholesterol-lowering medications.

CI, confidence interval; HR, hazard ratio; LPA, light physical activity; MVPA, moderate-to-vigorous physical activity; T2DM, type 2 diabetes mellitus.

**Supplementary Table S6. Subgroup analyses of associations of total volume of PA, MVPA, LPA, and sedentary behavior with incident T2DM stratified by age.**

| **Subgroups** | **Exposures** | **N** | **Incident rate**  **(per 1000 person-years)** | **Model 1^*^** | **Model 2^†^** | **Model 3^‡^** |
| --- | --- | --- | --- | --- | --- | --- |
|  |  |  |  | **HR (95% CI)** | **HR (95% CI)** | **HR (95% CI)** |
| **< 65 year** | **Total PA** |  |  |  |  |  |
|  | Low | 13,236 | 5.03 | 1 (Reference) | 1 (Reference) | 1 (Reference) |
|  | Moderate | 17,045 | 2.49 | 0.52 (0.45-0.61) | 0.66 (0.57-0.76) | 0.83 (0.72-0.97) |
|  | High | 20,802 | 1.45 | 0.31 (0.27-0.37) | 0.46 (0.39-0.55) | 0.64 (0.54-0.77) |
|  | **MVPA** |  |  |  |  |  |
|  | Not recommended | 27,139 | 3.91 | 1 (Reference) | 1 (Reference) | 1 (Reference) |
|  | Recommended | 23,944 | 1.37 | 0.34 (0.29-0.39) | 0.51 (0.44-0.60) | 0.64 (0.55-0.75) |
|  | **LPA** |  |  |  |  |  |
|  | Low | 16,582 | 3.71 | 1 (Reference) | 1 (Reference) | 1 (Reference) |
|  | Moderate | 17,111 | 2.22 | 0.65 (0.56-0.76) | 0.70 (0.60-0.82) | 0.87 (0.74-1.02) |
|  | High | 17,390 | 2.26 | 0.68 (0.59-0.80) | 0.71 (0.61-0.84) | 0.87 (0.74-1.03) |
|  | **Sedentary behavior** |  |  |  |  |  |
|  | Low | 17,296 | 1.53 | 1 (Reference) | 1 (Reference) | 1 (Reference) |
|  | Moderate | 17,059 | 2.29 | 1.43 (1.19-1.73) | 1.19 (0.99-1.44) | 1.16 (0.96-1.40) |
|  | High | 16,728 | 4.39 | 2.56 (2.16-3.04) | 1.71 (1.42-2.06) | 1.44 (1.19-1.74) |
|  | **Grip strength** |  |  |  |  |  |
|  | Normal | 48,274 | 2.58 | 1 (Reference) | 1 (Reference) | 1 (Reference) |
|  | Low | 2,809 | 5.00 | 1.92 (1.55-2.37) | 1.46 (1.18-1.81) | 1.40 (1.13-1.74) |
|  | **Walk pace** |  |  |  |  |  |
|  | Brisk | 25,906 | 9.21 | 1 (Reference) | 1 (Reference) | 1 (Reference) |
|  | Steady average | 23,248 | 3.66 | 2.57 (2.22-2.98) | 1.60 (1.38-1.87) | 1.42 (1.22-1.66) |
|  | Slow | 1,929 | 1.41 | 6.65 (5.35-8.27) | 2.41 (1.92-3.04) | 2.19 (1.74-2.76) |
|  | **Musle mass** |  |  |  |  |  |
|  | Normal | 31,485 | 2.11 | 1 (Reference) | 1 (Reference) | 1 (Reference) |
|  | Mild to moderate deficit | 17,700 | 3.31 | 1.40 (1.23-1.60) | 1.23 (1.07-1.41) | 1.29 (1.12-1.48) |
|  | Severe deficit | 1,898 | 7.30 | 2.63 (2.10-3.30) | 1.37 (1.08-1.73) | 1.25 (0.99-1.58) |
| **≥ 65 year** | **Total PA** |  |  |  |  |  |
|  | Low | 16,632 | 7.86 | 1 (Reference) | 1 (Reference) | 1 (Reference) |
|  | Moderate | 12,792 | 4.12 | 0.56 (0.49-0.63) | 0.68 (0.60-0.77) | 0.76 (0.67-0.86) |
|  | High | 9,025 | 2.70 | 0.38 (0.32-0.44) | 0.54 (0.46-0.64) | 0.63 (0.53-0.75) |
|  | **MVPA** |  |  |  |  |  |
|  | Not recommended | 26,768 | 6.38 | 1 (Reference) | 1 (Reference) | 1 (Reference) |
|  | Recommended | 11,681 | 3.11 | 0.45 (0.40-0.52) | 0.64 (0.56-0.74) | 0.72 (0.62-0.83) |
|  | **LPA** |  |  |  |  |  |
|  | Low | 13,262 | 6.93 | 1 (Reference) | 1 (Reference) | 1 (Reference) |
|  | Moderate | 12,733 | 4.89 | 0.78 (0.69-0.88) | 0.89 (0.78-1.01) | 0.89 (0.78-1.01) |
|  | High | 12,454 | 4.23 | 0.73 (0.64-0.83) | 0.86 (0.74-0.99) | 0.87 (0.75-1.00) |
|  | **Sedentary behavior** |  |  |  |  |  |
|  | Low | 12,548 | 3.26 | 1 (Reference) | 1 (Reference) | 1 (Reference) |
|  | Moderate | 12,785 | 4.71 | 1.34 (1.15-1.56) | 1.12 (0.96-1.31) | 1.06 (0.91-1.24) |
|  | High | 13,116 | 8.11 | 2.12 (1.84-2.44) | 1.33 (1.14-1.56) | 1.27 (1.09-1.50) |
|  | **Grip strength** |  |  |  |  |  |
|  | Normal | 34,090 | 5.32 | 1 (Reference) | 1 (Reference) | 1 (Reference) |
|  | Low | 4,359 | 5.78 | 1.16 (0.98-1.36) | 0.99 (0.84-1.16) | 0.93 (0.79-1.10) |
|  | **Walk pace** |  |  |  |  |  |
|  | Brisk | 15,702 | 13.88 | 1 (Reference) | 1 (Reference) | 1 (Reference) |
|  | Steady average | 20,541 | 5.97 | 1.70 (1.51-1.93) | 1.23 (1.08-1.39) | 1.14 (1.01-1.30) |
|  | Slow | 2,206 | 3.48 | 4.04 (3.39-4.80) | 1.82 (1.52-2.19) | 1.63 (1.35-1.96) |
|  | **Musle mass** |  |  |  |  |  |
|  | Normal | 16,499 | 4.20 | 1 (Reference) | 1 (Reference) | 1 (Reference) |
|  | Mild to moderate deficit | 18,187 | 5.69 | 1.17 (1.03-1.31) | 1.11 (0.99-1.25) | 1.17 (1.03-1.32) |
|  | Severe deficit | 3,763 | 9.08 | 1.54 (1.30-1.82) | 1.14 (0.96-1.34) | 1.10 (0.93-1.30) |

*Model 1 was adjusted for age at the time of accelerometer wearing and sex.

†Model 2 was adjusted for age at the time of accelerometer wearing, sex, ethnicity, Townsend deprivation index, recruitment center, education level, the season of accelerometer wearing, obesity status, healthy diet score, sleep duration, grip strength, smoking status, alcohol consumption, and HbA1c.

‡Model 3 was adjusted for age at the time of accelerometer wearing, sex, ethnicity, Townsend deprivation index, recruitment center, education level, the season of accelerometer wearing, obesity status, healthy diet score, sleep duration, grip strength, smoking status, alcohol consumption, HbA1c, hypertension, high cholesterol, history of coronary heart diseases, history of cancers, use of blood pressure-lowering medications, and use of cholesterol-lowering medications.

CI, confidence interval; HR, hazard ratio; LPA, light physical activity; MVPA, moderate-to-vigorous physical activity; T2DM, type 2 diabetes mellitus.

**Supplementary Table S7. Subgroup analyses of associations of total volume of PA, MVPA, LPA, and sedentary behavior with incident T2DM stratified by sex.**

| **Subgroups** | **Exposures** | **N** | **Incident rate**  **(per 1000 person-years)** | **Model 1^*^** | **Model 2^†^** | **Model 3^‡^** |
| --- | --- | --- | --- | --- | --- | --- |
|  |  |  |  | **HR (95% CI)** | **HR (95% CI)** | **HR (95% CI)** |
| **Female** | **Total PA** |  |  |  |  |  |
|  | Low | 15,605 | 4.49 | 1 (Reference) | 1 (Reference) | 1 (Reference) |
|  | Moderate | 17,508 | 2.07 | 0.49 (0.42-0.58) | 0.65 (0.56-0.77) | 0.76 (0.65-0.89) |
|  | High | 17,824 | 1.43 | 0.37 (0.31-0.44) | 0.61 (0.51-0.74) | 0.76 (0.63-0.92) |
|  | **MVPA** |  |  |  |  |  |
|  | Not recommended | 32,584 | 3.36 | 1 (Reference) | 1 (Reference) | 1 (Reference) |
|  | Recommended | 18,353 | 1.20 | 0.41 (0.34-0.48) | 0.67 (0.56-0.80) | 0.81 (0.67-0.97) |
|  | **LPA** |  |  |  |  |  |
|  | Low | 13,625 | 3.56 | 1 (Reference) | 1 (Reference) | 1 (Reference) |
|  | Moderate | 17,494 | 2.14 | 0.60 (0.51-0.70) | 0.71 (0.60-0.83) | 0.81 (0.68-0.96) |
|  | High | 19,818 | 2.28 | 0.63 (0.54-0.73) | 0.78 (0.66-0.92) | 0.86 (0.73-1.02) |
|  | **Sedentary behavior** |  |  |  |  |  |
|  | Low | 20,305 | 1.76 | 1 (Reference) | 1 (Reference) | 1 (Reference) |
|  | Moderate | 17,463 | 2.30 | 1.31 (1.10-1.55) | 1.03 (0.87-1.23) | 1.07 (0.90-1.28) |
|  | High | 13,169 | 4.22 | 2.40 (2.04-2.81) | 1.34 (1.12-1.61) | 1.24 (1.03-1.50) |
|  | **Grip strength** |  |  |  |  |  |
|  | Normal | 46,249 | 2.46 | 1 (Reference) | 1 (Reference) | 1 (Reference) |
|  | Low | 4,688 | 3.74 | 1.28 (1.05-1.56) | 1.04 (0.86-1.27) | 1.06 (0.87-1.30) |
|  | **Walk pace** |  |  |  |  |  |
|  | Brisk | 23,344 | 8.70 | 1 (Reference) | 1 (Reference) | 1 (Reference) |
|  | Steady average | 25,058 | 3.10 | 2.12 (1.81-2.48) | 1.27 (1.08-1.50) | 1.16 (0.98-1.37) |
|  | Slow | 2,535 | 1.37 | 5.70 (4.63-7.03) | 1.99 (1.59-2.49) | 1.75 (1.39-2.20) |
|  | **Musle mass** |  |  |  |  |  |
|  | Normal | 31,167 | 1.93 | 1 (Reference) | 1 (Reference) | 1 (Reference) |
|  | Mild to moderate deficit | 18,414 | 3.34 | 1.59 (1.38-1.82) | 1.10 (0.96-1.27) | 1.26 (1.09-1.46) |
|  | Severe deficit | 1,356 | 7.20 | 3.29 (2.53-4.27) | 1.09 (0.82-1.43) | 1.14 (0.86-1.51) |
| **Male** | **Total PA** |  |  |  |  |  |
|  | Low | 15,605 | 4.49 | 1 (Reference) | 1 (Reference) | 1 (Reference) |
|  | Moderate | 17,508 | 2.07 | 0.49 (0.42-0.58) | 0.65 (0.56-0.77) | 0.76 (0.65-0.89) |
|  | High | 17,824 | 1.43 | 0.37 (0.31-0.44) | 0.61 (0.51-0.74) | 0.76 (0.63-0.92) |
|  | **MVPA** |  |  |  |  |  |
|  | Not recommended | 32,584 | 3.36 | 1 (Reference) | 1 (Reference) | 1 (Reference) |
|  | Recommended | 18,353 | 1.20 | 0.41 (0.34-0.48) | 0.67 (0.56-0.80) | 0.81 (0.67-0.97) |
|  | **LPA** |  |  |  |  |  |
|  | Low | 13,625 | 3.56 | 1 (Reference) | 1 (Reference) | 1 (Reference) |
|  | Moderate | 17,494 | 2.14 | 0.60 (0.51-0.70) | 0.71 (0.60-0.83) | 0.81 (0.68-0.96) |
|  | High | 19,818 | 2.28 | 0.63 (0.54-0.73) | 0.78 (0.66-0.92) | 0.86 (0.73-1.02) |
|  | **Sedentary behavior** |  |  |  |  |  |
|  | Low | 20,305 | 1.76 | 1 (Reference) | 1 (Reference) | 1 (Reference) |
|  | Moderate | 17,463 | 2.30 | 1.31 (1.10-1.55) | 1.03 (0.87-1.23) | 1.07 (0.90-1.28) |
|  | High | 13,169 | 4.22 | 2.40 (2.04-2.81) | 1.34 (1.12-1.61) | 1.24 (1.03-1.50) |
|  | **Grip strength** |  |  |  |  |  |
|  | Normal | 46,249 | 2.46 | 1 (Reference) | 1 (Reference) | 1 (Reference) |
|  | Low | 4,688 | 3.74 | 1.28 (1.05-1.56) | 1.04 (0.86-1.27) | 1.06 (0.87-1.30) |
|  | **Walk pace** |  |  |  |  |  |
|  | Brisk | 23,344 | 8.70 | 1 (Reference) | 1 (Reference) | 1 (Reference) |
|  | Steady average | 25,058 | 3.10 | 2.12 (1.81-2.48) | 1.27 (1.08-1.50) | 1.16 (0.98-1.37) |
|  | Slow | 2,535 | 1.37 | 5.70 (4.63-7.03) | 1.99 (1.59-2.49) | 1.75 (1.39-2.20) |
|  | **Musle mass** |  |  |  |  |  |
|  | Normal | 31,167 | 1.93 | 1 (Reference) | 1 (Reference) | 1 (Reference) |
|  | Mild to moderate deficit | 18,414 | 3.34 | 1.59 (1.38-1.82) | 1.10 (0.96-1.27) | 1.26 (1.09-1.46) |
|  | Severe deficit | 1,356 | 7.20 | 3.29 (2.53-4.27) | 1.09 (0.82-1.43) | 1.14 (0.86-1.51) |

*Model 1 was adjusted for age at the time of accelerometer wearing and sex.

†Model 2 was adjusted for age at the time of accelerometer wearing, sex, ethnicity, Townsend deprivation index, recruitment center, education level, the season of accelerometer wearing, obesity status, healthy diet score, sleep duration, grip strength, smoking status, alcohol consumption, and HbA1c.

‡Model 3 was adjusted for age at the time of accelerometer wearing, sex, ethnicity, Townsend deprivation index, recruitment center, education level, the season of accelerometer wearing, obesity status, healthy diet score, sleep duration, grip strength, smoking status, alcohol consumption, HbA1c, hypertension, high cholesterol, history of coronary heart diseases, history of cancers, use of blood pressure-lowering medications, and use of cholesterol-lowering medications.

CI, confidence interval; HR, hazard ratio; LPA, light physical activity; MVPA, moderate-to-vigorous physical activity; T2DM, type 2 diabetes mellitus.

**Supplementary Table S8. Subgroup analyses of associations of total volume of PA, MVPA, LPA, and sedentary behavior with incident T2DM stratified by obese status.**

| **Subgroups** | **Exposures** | **N** | **Incident rate**  **(per 1000 person-years)** | **Model 1^*^** | **Model 2^†^** | **Model 3^‡^** |
| --- | --- | --- | --- | --- | --- | --- |
|  |  |  |  | **HR (95% CI)** | **HR (95% CI)** | **HR (95% CI)** |
| **Overweight** | **Total PA** |  |  |  |  |  |
|  | Low | 12,923 | 5.54 | 1 (Reference) | 1 (Reference) | 1 (Reference) |
|  | Moderate | 12,806 | 3.09 | 0.63 (0.55-0.74) | 0.64 (0.55-0.75) | 0.72 (0.62-0.84) |
|  | High | 11,019 | 2.08 | 0.47 (0.39-0.56) | 0.45 (0.38-0.55) | 0.61 (0.50-0.73) |
|  | **MVPA** |  |  |  |  |  |
|  | Not recommended | 22,895 | 4.39 | 1 (Reference) | 1 (Reference) | 1 (Reference) |
|  | Recommended | 13,853 | 2.39 | 0.57 (0.49-0.67) | 0.61 (0.52-0.71) | 0.73 (0.62-0.85) |
|  | **LPA** |  |  |  |  |  |
|  | Low | 12,744 | 4.36 | 1 (Reference) | 1 (Reference) | 1 (Reference) |
|  | Moderate | 12,324 | 3.52 | 0.89 (0.76-1.03) | 0.88 (0.75-1.03) | 0.88 (0.75-1.03) |
|  | High | 11,680 | 2.97 | 0.79 (0.67-0.94) | 0.75 (0.63-0.89) | 0.83 (0.70-0.99) |
|  | **Sedentary behavior** |  |  |  |  |  |
|  | Low | 11,147 | 2.54 | 1 (Reference) | 1 (Reference) | 1 (Reference) |
|  | Moderate | 12,651 | 3.07 | 1.14 (0.95-1.37) | 1.13 (0.94-1.36) | 1.13 (0.94-1.37) |
|  | High | 12,950 | 5.14 | 1.78 (1.50-2.11) | 1.67 (1.39-2.01) | 1.58 (1.31-1.91) |
|  | **Grip strength** |  |  |  |  |  |
|  | Normal | 33,895 | 3.52 | 1 (Reference) | 1 (Reference) | 1 (Reference) |
|  | Low | 2,853 | 4.94 | 1.26 (1.02-1.57) | 1.12 (0.91-1.39) | 1.12 (0.91-1.40) |
|  | **Walk pace** |  |  |  |  |  |
|  | Brisk | 16,108 | 7.34 | 1 (Reference) | 1 (Reference) | 1 (Reference) |
|  | Steady average | 19,278 | 4.14 | 1.45 (1.26-1.67) | 1.32 (1.15-1.53) | 1.16 (1.01-1.34) |
|  | Slow | 1,362 | 2.72 | 2.45 (1.88-3.19) | 1.81 (1.38-2.38) | 1.52 (1.15-2.01) |
|  | **Musle mass** |  |  |  |  |  |
|  | Normal | 23,937 | 2.89 | 1 (Reference) | 1 (Reference) | 1 (Reference) |
|  | Mild to moderate deficit | 11,328 | 4.76 | 1.26 (1.09-1.45) | 1.14 (0.99-1.32) | 1.12 (0.97-1.29) |
|  | Severe deficit | 1,483 | 7.12 | 1.46 (1.12-1.89) | 1.16 (0.89-1.51) | 1.13 (0.87-1.48) |
| **Normal** | **Total PA** |  |  |  |  |  |
|  | Low | 8,670 | 2.09 | 1 (Reference) | 1 (Reference) | 1 (Reference) |
|  | Moderate | 11,916 | 1.03 | 0.58 (0.44-0.77) | 0.63 (0.48-0.83) | 0.84 (0.63-1.13) |
|  | High | 15,612 | 0.81 | 0.54 (0.41-0.72) | 0.57 (0.43-0.76) | 0.77 (0.57-1.04) |
|  | **MVPA** |  |  |  |  |  |
|  | Not recommended | 18,135 | 1.62 | 1 (Reference) | 1 (Reference) | 1 (Reference) |
|  | Recommended | 18,063 | 0.74 | 0.50 (0.39-0.64) | 0.55 (0.43-0.71) | 0.63 (0.49-0.82) |
|  | **LPA** |  |  |  |  |  |
|  | Low | 10,251 | 1.61 | 1 (Reference) | 1 (Reference) | 1 (Reference) |
|  | Moderate | 12,336 | 1.00 | 0.69 (0.52-0.91) | 0.71 (0.53-0.94) | 0.77 (0.58-1.03) |
|  | High | 13,611 | 1.02 | 0.74 (0.56-0.98) | 0.72 (0.53-0.96) | 0.80 (0.59-1.08) |
|  | **Sedentary behavior** |  |  |  |  |  |
|  | Low | 15,260 | 0.79 | 1 (Reference) | 1 (Reference) | 1 (Reference) |
|  | Moderate | 12,063 | 1.27 | 1.51 (1.13-2.01) | 1.48 (1.10-1.98) | 1.35 (1.01-1.82) |
|  | High | 8,875 | 1.75 | 1.91 (1.42-2.55) | 1.66 (1.20-2.30) | 1.47 (1.05-2.05) |
|  | **Grip strength** |  |  |  |  |  |
|  | Normal | 33,441 | 1.09 | 1 (Reference) | 1 (Reference) | 1 (Reference) |
|  | Low | 2,757 | 2.28 | 1.75 (1.26-2.43) | 1.43 (1.03-1.99) | 1.29 (0.92-1.81) |
|  | **Walk pace** |  |  |  |  |  |
|  | Brisk | 21,392 | 3.70 | 1 (Reference) | 1 (Reference) | 1 (Reference) |
|  | Steady average | 14,084 | 1.62 | 1.75 (1.38-2.22) | 1.52 (1.19-1.93) | 1.29 (1.00-1.65) |
|  | Slow | 722 | 0.81 | 3.97 (2.42-6.51) | 2.64 (1.59-4.38) | 1.80 (1.04-3.12) |
|  | **Musle mass** |  |  |  |  |  |
|  | Normal | 17,708 | 0.82 | 1 (Reference) | 1 (Reference) | 1 (Reference) |
|  | Mild to moderate deficit | 16,601 | 1.41 | 1.04 (0.79-1.37) | 0.98 (0.74-1.30) | 1.14 (0.85-1.53) |
|  | Severe deficit | 1,889 | 2.64 | 1.15 (0.74-1.79) | 0.97 (0.62-1.51) | 1.24 (0.78-1.98) |

*Model 1 was adjusted for age at the time of accelerometer wearing and sex.

†Model 2 was adjusted for age at the time of accelerometer wearing, sex, ethnicity, Townsend deprivation index, recruitment center, education level, the season of accelerometer wearing, obesity status, healthy diet score, sleep duration, grip strength, smoking status, alcohol consumption, and HbA1c.

‡Model 3 was adjusted for age at the time of accelerometer wearing, sex, ethnicity, Townsend deprivation index, recruitment center, education level, the season of accelerometer wearing, obesity status, healthy diet score, sleep duration, grip strength, smoking status, alcohol consumption, HbA1c, hypertension, high cholesterol, history of coronary heart diseases, history of cancers, use of blood pressure-lowering medications, and use of cholesterol-lowering medications.

CI, confidence interval; HR, hazard ratio; LPA, light physical activity; MVPA, moderate-to-vigorous physical activity; T2DM, type 2 diabetes mellitus.
